# Magnetic/photothermal dual-driven micro/nanorobots for synergistic NO-mediated photothermal thrombolysis

**DOI:** 10.1016/j.mtbio.2026.103179

**Published:** 2026-04-30

**Authors:** Wenjia Kang, Jinhua Li, Song Li, Yingting Yang, Shanqing Gao, Shuangying Wei, Zdenek Sofer, Jiatao Zhang, Huaijuan Zhou

**Affiliations:** a School of Materials Science and Engineering, School of Interdisciplinary Science, Beijing Institute of Technology, Beijing, 100081, China; b Key Laboratory of Medical Molecule Science and Pharmaceutical Engineering, Ministry of Industry and Information Technology, Beijing Key Laboratory of Intelligent Molecular Materials and High-throughput Manufacturing, School of Chemistry and Chemical Engineering, Beijing Institute of Technology, Beijing, 100081, China; c Department of Industrial Chemistry, University of Bologna, Campus Navile, Via Piero Gobetti 85, Bologna, 40139, Italy; d Department of Inorganic Chemistry, University of Chemistry and Technology Prague, 16628, Czech Republic

**Keywords:** Micro/nanorobots, Photothermal therapy, Dual-driven, Thrombosis treatment, Nitric oxide release

## Abstract

Thrombotic vascular obstruction, a leading cause of cardiovascular/cerebrovascular events and global mortality, poses a severe threat to human health, while conventional thrombolytic agents suffer from inherent limitations, including short half-lives, poor targeting, low utilization efficiency, and suboptimal therapeutic outcomes. Nitric oxide (NO)-supported gas therapy exhibits significant potential in synergistic thrombolysis but is hindered by its ultra-short half-life and challenges in targeted delivery and spatiotemporal control. Herein, we developed dual-driven rGO@Fe_3_O_4_-βCD-BNN6 micro/nanorobots for NO-mediated targeted photothermal thrombus ablation, with the advantages of site-specific accumulation, controlled therapeutic agent release, and enhanced therapeutic specificity. Reduced graphene oxide (rGO) served as the core photothermal agent with excellent photothermal conversion efficiency (44.2%), while Fe_3_O_4_ nanoparticles endowed the micro/nanorobots with magnetic responsiveness for precise directional targeting. β-Cyclodextrin (βCD) enhanced the loading capacity and biocompatibility of the NO donor BNN6 (N,N′-di-sec-butyl-N,N′-dinitroso-1,4-phenylene diamine), which underwent photothermal-induced thermal decomposition to release NO in situ, disrupting fibrin networks and synergistically boosting thrombolysis. The micro/nanorobots achieved a remarkable thrombolytic efficacy of up to 88.8%, significantly outperforming conventional drugs. Comprehensive hemolysis and cytotoxicity assays confirmed their excellent biocompatibility. This dual-driven, photothermal-gas synergistic micro/nanorobotic platform provides a novel and safe strategy to overcome the limitations of traditional thrombolysis, paving the way for advancing micro/nanorobotic thrombotic intervention.

## Highlights

• Development of magnetic-optical actuated micro/nanorobots enabling precise spatiotemporal motion control.
• Photothermally triggered on-demand nitric oxide (NO) release for targeted thrombolytic therapy.
• Exceptional thrombolysis efficiency (up to 88.8%) achieved via synergistic actuation-NO release coupling.

## Graphical abstract

Dual-driven (magnetic/photothermal) rGO@Fe_3_O_4_-βCD-BNN6 micro/nanorobots for targeted thrombolysis: leveraging NIR laser irradiation to trigger both photothermal thrombus ablation and photothermally induced decomposition of BNN6 for NO release, thereby achieving synergistic therapeutic effects.

**Figure:**
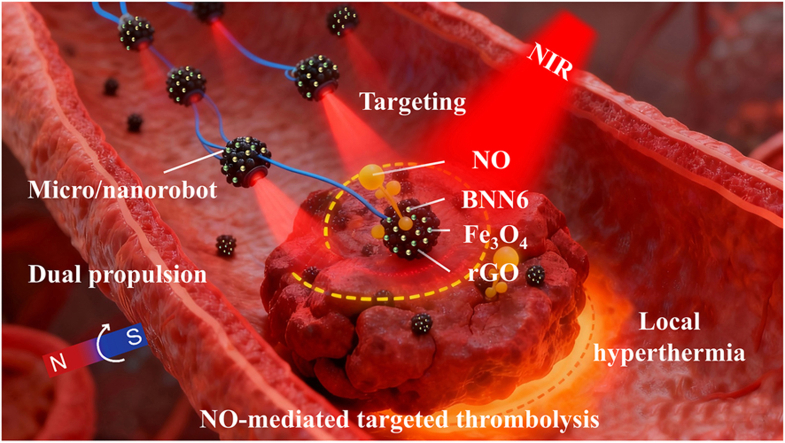

## 1 Introduction

Thrombotic diseases, characterized by abnormal blood coagulation leading to vascular obstruction, have emerged as the leading cause of global mortality, as they trigger severe cardiovascular and cerebrovascular events [[1], [2], [3], [4]]. Clinically, thrombolytic agents such as tissue plasminogen activator (tPA) [5], urokinase (UK) [6], and streptokinase (SK) remain the first-line therapeutic options, often supplemented with anticoagulants to prevent recurrence [7]. However, these conventional drugs suffer from inherent limitations, including short half-lives and poor targeting capabilities, which result in low drug utilization, high clinical costs, and suboptimal therapeutic outcomes [8,9]. This urgent clinical dilemma highlights the imperative need for innovative, targeted therapeutic strategies to overcome the drawbacks of traditional treatments, particularly in the realm of physical-chemical synergistic therapy.

Gas therapy (GT) is a promising and biocompatible therapeutic strategy with high efficacy [[10], [11], [12], [13], [14]]. Among gasotransmitters, nitric oxide (NO) is particularly pivotal in synergistic photothermal thrombolysis, as it regulates vascular endothelial function, inhibits platelet aggregation, and mitigates oxidative stress in the thrombus microenvironment [[15], [16], [17], [18]]. Notably, the local hyperthermia from photothermal therapy (PTT) not only suppresses thrombus progression but also accelerates NO diffusion, synergistically improving the thrombus microenvironment and therapeutic efficacy. However, NO's ultra-short half-life (<5 s) severely hinders its clinical translation. Achieving efficient targeted delivery and precise spatiotemporal control of NO (especially via internal/external stimuli) remains a critical challenge in thrombotic therapy [19].

Micro/nanorobots (MNRs), with sizes ranging from micrometers to nanometers, have emerged as a revolutionary platform for targeted thrombosis therapy due to their ability to execute specific tasks at the microscale [[20], [21], [22], [23], [24], [25], [26]]. Powered by diverse propulsion modalities, including magnetic fields [27], light fields [28], ultrasound fields [29,30], and chemical fuels [31], MNRs offer enhanced controllability and maneuverability, addressing the targeting limitations of conventional therapeutic agents. For thrombosis therapy, near-infrared light (NIR) is particularly advantageous as it can simultaneously serve as a propulsion source and therapeutic stimulus [[32], [33], [34], [35]]. Notably, integrating photothermal therapy with magnetic/photothermal-driven propulsion into MNRs yields a superior thrombolytic paradigm: compared to single-mode systems, this dual-drive configuration enables deeper tissue penetration and more precise targeting, thereby augmenting therapeutic efficacy. Furthermore, MNRs can be engineered as intelligent carriers for gasotransmitters, offering a feasible approach to addressing the challeges associated with NO delivery and spatiotemporal controlled realease.

The performance of MNRs in thrombosis therapy is highly dependent on the selection of constituent materials. Integrating MNRs with photothermal materials confers photothermal properties or photothermal actuation capabilities, markedly enhancing the therapeutic targeting and precision. Common photothermal materials include Au nanoparticles [36], carbon-based materials [37], organic compounds [29,30], and two-dimensional materials [38]. Among these, a variety of biodegradable photothermal candidates have been reported in recent studies [[39], [40], [41]]. Phosphorus-based materials, such as black phosphorus and purple phosphorus, tend to degrade rapidly in physiological environments, resulting in sufficient photothermal stability [[42], [43], [44]]. In contrast, manganese-based materials are limited by low photothermal conversion efficiency and potential toxicity associated with metal ion realease [45]. Notably, graphene (a carbon-based material) stands out for its exceptional photothermal effects, high specific surface area, and biocompatibility [46] with its sp^2^/sp hybridized carbon structure enabling diverse chemical modifications and adjustable architectures, making it an ideal candidate for MNR construction [47,48]. Graphene oxide (GO) and its reduced form (rGO) have thus been widely explored in photothermal MNRs for biomedical applications [[49], [50], [51], [52]].

Synergistic application of photothermal therapy and GT holds substantial promise for enhanced thrombolysis. For instance, NO can effectively dissolve blood clots and prevent recurrence by activating endothelial nitric oxide synthase to improve microcirculation at the thrombosis site [53], generating mechanical forces to directly disrupt clots and facilitating the penetration of MNRs into thrombus matrices [17,[54], [55], [56]]. Most traditional NO donors, such as azine diol salts (DEA/NO) [57] or S-nitrosothiols (GSNO) [58], are prone to non-specific decomposition under physiological conditions. This non-specific decomposition leads to poor stability and premature release of NO, thereby limiting their therapeutic efficacy. Notably, NO donors such as BNN6 can undergo thermal decomposition under NIR-induced photothermal heating to achieve controlled NO release, ensuring precise spatiotemporal regulation and minimizing off-target side effects [59]. Integrating such NO donors into photothermal-magnetic dual-driven MNRs thus enables synergistic photothermal-gas therapy for thrombosis, which is expected to outperform standalone photothermal or NO therapy.

**Scheme 1:**
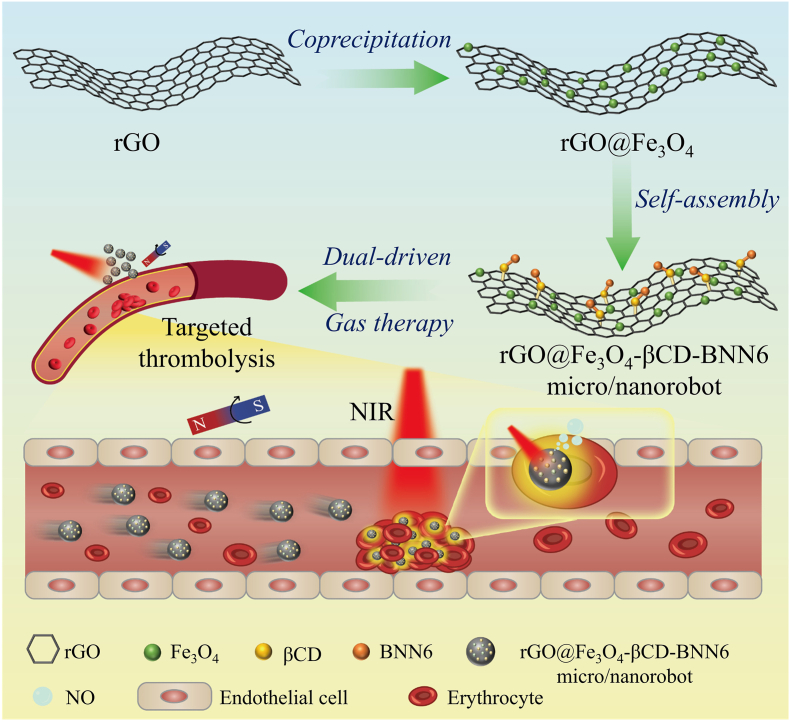
Schematic diagram depicting the synthetic route of rGO@Fe_3_O_4_-βCD-BNN6 micro/nanorobots and their application for targeted and controlled thrombolysis through the integration of photothermal-magnetic dual-driven propulsion effects and NO-mediated gas therapy.

Herein, we developed dual-driven (magnetic/photothermal) rGO@Fe_3_O_4_-βCD-BNN6 micro/nanorobots for NO-mediated photothermal thrombosis therapy (Scheme 1). In this system, rGO serves as the primary photothermal agent, achieving a rapid temperature increase of 26 °C within 10 min with a high photothermal conversion efficiency of 44.2%, and it also generates photothermal swimming forces to induce micro/nanorobot aggregation under NIR irradiation, further boosting photothermal performance. Fe_3_O_4_ particles loaded onto rGO endowed the micro/nanorobots with magnetic responsiveness, enabling directional movement under an external magnetic field for targeted deep therapy. β-cyclodextrin (βCD) is introduced to improve the loading capacity and biocompatibility of BNN6 (a NO donor), which undergoes in-situ thermal decomposition triggered by the photothermal effect of rGO to release NO, thereby enhancing gas-assisted thrombolysis. Benefiting from its superior photothermal performance, precise magnetic targeting, and controlled NO release, the rGO@Fe_3_O_4_-βCD-BNN6 micro/nanorobots exhibit remarkable thrombolytic efficacy (up to 88.8%). In addition, hemolysis and cytotoxicity assays confirm their excellent hemocompatibility and biocompatibility. This work establishes a dual-driven, photothermal-gas synergistic targeted therapeutic MNR platform for thrombosis therapy, and lays a solid foundation for advancing MNR-based thrombotic intervention.

## 2 Experimental section

### 2.1 Materials

Iron (II) sulfate heptahydrate (FeSO_4_∙7H_2_O), iron (III) chloride hexahydrate (FeCl_3_∙6H_2_O), β-cyclodextrin, dimethyl sulfoxide (DMSO), Tween-80, and nitric oxide (NO) assay kit were purchased from Titan Scientific Co., Ltd. (Shanghai, China). Sodium hydroxide (NaOH) was provided by Macklin Biochemical Co., Ltd. (Shanghai, China). BNN6 was obtained from APE × BIO Technology LLC (Shanghai, China). Heparin sodium-anticoagulated rabbit blood was supplied by SenBeiJia Biological Technology Co., Ltd. (Nanjing, China). 10 × phosphate-buffered saline (10 × PBS) was purchased from Solarbio Science & Technology Co., Ltd. (Beijing, China). Urokinase (UK) was obtained from Bioss Biotechnology Co., Ltd. (Beijing, China). Cell Counting Kit-8 (CCK-8) was provided by Titan Scientific Co., Ltd. (Shanghai, China), and Calcein/PI Cell Viability and Cytotoxicity Assay Kit was purchased from Biyuntian Biological Co., Ltd. (Shanghai, China). NO scavenger was obtained from Beyotime Biotech Inc. (Shanghai, China). Activated Partial Thromboplastin Time (APTT) Test Kit was provided by Beijing leagene biotech.co.,ltd. (Beijing, China). All commercial reagents were used directly without further purification. rGO was offered by Prof. Zdenek Sofer.

### 2.2 Synthesis of rGO@Fe_3_O_4_

rGO@Fe_3_O_4_ was synthesized via the coprecipitation method, following these steps: First, 10 mg of rGO was dispersed in 20 mL of deionized water (H_2_O) and homogenized by ultrasonic treatment to ensure complete dispersion. Separately, 24.015 mg of FeSO_4_∙7H_2_O and 46.651 mg of FeCl_3_∙6H_2_O were dissolved in 40 mL of H_2_O to form a homogeneous metal ion solution. The rGO dispersion was then added dropwise to the metal ion solution, and the mixture was stirred continuously for 1 h to achieve uniform mixing.

Subsequently, 1 M NaOH solution was added to the above mixture until the pH reached 10. Simultaneously, the mixture was heated to 180 °C and stirred at this temperature for 1 h to initiate the coprecipitation reaction. After the reaction, the mixture was allowed to stand for phase separation, followed by centrifugation at 8000 rpm for 5 min. This centrifugation step was repeated three times to purify the product. Finally, the collected precipitate was vacuum-dried at 60 °C, and the resulting solid was ground into a fine powder to obtain rGO@Fe_3_O_4_ powder.

### 2.3 Synthesis of rGO@Fe_3_O_4_-βCD-BNN6 micro/nanorobots

First, 10 mg of rGO@Fe_3_O_4_ (synthesized as described above), 25 mg of β-cyclodextrin (βCD), and 20 mL of deionized water were mixed and stirred at room temperature for 12 h to enable sufficient interaction between rGO@Fe_3_O_4_ and βCD. The mixture was then washed with deionized water and centrifuged three times to remove unbound βCD. Finally, the collected product was vacuum-dried at 60 °C to obtain rGO@Fe_3_O_4_-βCD powder.

For BNN6 loading, 5 mg of rGO@Fe_3_O_4_-βCD was dispersed in 10 mL of deionized water and stirred to form a homogeneous dispersion. Separately, 5 mg of BNN6 was dissolved in 10 mL of DMSO to prepare a BNN6 solution. This BNN6 solution was then added dropwise to the rGO@Fe_3_O_4_-βCD dispersion, and the mixture was stirred in the dark for 12 h (to avoid light-induced BNN6 degradation), followed by standing for 3 h to facilitate self-assembly. The resulting mixture was centrifuged and washed three times to eliminate free BNN6 and residual DMSO, then freeze-dried at −80 °C to obtain rGO@Fe_3_O_4_-βCD-BNN6 micro/nanorobots.

### 2.4 Materials characterization and performance evaluation

For structural and compositional characterization of the samples, X-ray diffraction (XRD) analysis was performed using a Rigaku ULTIMA IV X-ray Powder Diffractometer to determine crystal phases; Fourier Transform Infrared Spectroscopy (FTIR) measurements were conducted with a Thermo Scientific Nicolet iS50 FTIR spectrometer to analyze chemical bonds and functional groups; and UV-Visible Spectrophotometry (UV-Vis) was carried out using a Beijing Puxi TU-1901 Double-Beam UV-Vis spectrophotometer to evaluate optical absorption properties.

For microstructural characterization, scanning electron microscopy (SEM) was employed to observe the surface morphology of the samples, using a Zeiss Supra 55 SEM; transmission electron microscopy (TEM) was used to characterize the internal microstructure and particle distribution, with a Tecnai G2 F30 TEM from Field Electron and Ion Company. Meanwhile, the particle size of the materials was determined via a Malvern Zetasizer Ultra.

For experimental auxiliary equipment, an 808 nm infrared fiber-coupled laser (model FC-808-5000-MM, Shanghai Xilong Optoelectronic Technology Co., Ltd.) was utilized for photothermal stimulation; an infrared thermal imager (model Testo 875 Pro, Testo SE & Co. KGaA) was used to monitor solution temperature changes during photothermal treatment; a Nikon Eclipse Ti2 inverted fluorescence microscope was employed to capture videos for recording the motility of the micro/nanorobots; and a full-wavelength light absorption spectrophotometer (model Readmax 1900, Shanghai Flash Bio-Tech Co., Ltd.) was used to measure the absorbance of solutions.

### 2.5 Motion behavior of rGO@Fe_3_O_4_

To evaluate the magnetic-driven motion of rGO@Fe_3_O_4_, an appropriate amount of Tween-80 solution was added to the rGO@Fe_3_O_4_ dispersion, and the mixture was thoroughly homogenized via ultrasonic treatment. The resulting dispersion was dropped onto a hydrophilic-treated glass slide, and a rotating magnet was applied to provide a rotating magnetic field. The motion behavior of the micro/nanorobots was observed and recorded under an inverted fluorescence microscope. For the light-driven motion test, an 808 nm NIR laser was used as the illumination source, and the light-driven behavior of the micro/nanorobots was similarly observed under the inverted fluorescence microscope. Additionally, 808 nm NIR light sources with different intensities were employed to investigate the effect of light intensity on the micro/nanorobots’ motion speed. The motion trajectories of the micro/nanorobot particles were analyzed using Fiji software.

### 2.6 NIR photothermal property

For photothermal performance comparison, 2 mL of Fe_3_O_4_, rGO, and rGO@Fe_3_O_4_ solutions (with different concentrations) were separately added to test tubes, and each solution was irradiated with a 2.0 W/cm^2^ 808 nm NIR laser for 10 min; an infrared thermal imager was used to record the solution temperature every 15 s. To investigate the effect of laser intensity on photothermal properties, the same test was repeated using 808 nm NIR lasers with different intensities. For photothermal stability verification, the rGO@Fe_3_O_4_ solution was irradiated with NIR laser for 10 min, followed by laser shutdown to allow cooling; this on/off cycle of NIR irradiation was repeated three times, with temperature data recorded throughout.

To calculate the photothermal conversion efficiency (η) of rGO@Fe_3_O_4_, both the rGO@Fe_3_O_4_ solution and deionized water (as a control) were irradiated with a 2.0 W/cm^2^ 808 nm NIR laser for 10 min, then cooled to room temperature. *η* was determined using Equation (1) [59]:

(1) $$\eta =\frac{hS\left(T_{\max}-T_{sur}\right)-Q_{Dis}}{I\left(1-10^{-A_{\lambda }}\right)}$$

where *h* is the heat transfer coefficient, *S* is the surface area of the test fixture, *T*_max_ is the maximum temperature of the rGO@Fe_3_O_4_ solution, *T*_sur_ is the ambient temperature, *I* is the NIR laser intensity (2.0 W/cm^2^), and *A*_λ_ is the absorbance of the rGO@Fe_3_O_4_ solution at 808 nm. *Q*_Dis_ is the heat loss from solvent and fixture optical losses (measured using H_2_O via Equation (2)). The *hS* value was calculated using Equation (3):

(2) $$Q_{Dis}=\frac{m_{d}C_{d}\left(T_{\max}-T_{sur}\right)}{t}$$

(3) $$\tau_{s}=\frac{m_{d}C_{d}}{hS}$$

where *t* is the time when the temperature difference of H_2_O reaches its maximum, and *T*_max_ here refers to the maximum temperature of H_2_O. *m*_d_ is the mass of the solvent (2.0 g). *C*_d_ is the specific heat capacity of H_2_O (4.2 J g^−1^ ^o^C^−1^), and *τ*_s_ is the time constant (derived from Equation (4)). Equation (4) is defined as:

(4) $$t'=-\tau_{s}\;\ln\left(\theta \right)=-\tau_{s}\;\ln\left(\frac{T-T_{sur}}{T_{\max}-T_{sur}}\right)$$

where *T* is the actual temperature at each time point, *t'* is the cooling time, and *τ*_s_ is obtained from the slope of the fitted curve (with cooling time *t* on the y-axis and -*ln*(θ) on the x-axis, as shown in Fig. S10).

### 2.7 Measurement of NO generation

The NO generation capacity of rGO@Fe_3_O_4_-βCD-BNN6 micro/nanorobots was evaluated using the Griess assay. For the initial NO generation test, 3 mL of deionized water, 0.5 mg/mL rGO@Fe_3_O_4_ solution, and 0.5 mg/mL rGO@Fe_3_O_4_-βCD-BNN6 micro/nanorobots were each irradiated with a 2.0 W/cm^2^ 808 nm NIR laser for 1 h; 100 μL of supernatant was collected from each sample every 10 min for subsequent analysis. To assess the light-controlled NO release performance of rGO@Fe_3_O_4_-βCD-BNN6 micro/nanorobots, the NIR laser was turned on and off at 10-min intervals during irradiation, with 100 μL of supernatant collected after each on/off cycle and tested via the same Griess assay protocol. For quantitative NO determination, standard solutions of sodium nitrite (NaNO_2_) with different concentrations were prepared, and their optical density at 540 nm (OD_540nm_) was measured to establish a standard curve (Fig. S12); the OD_540nm_ of the collected test supernatants was then measured, and the corresponding NO content was calculated by referencing the standard curve.

### *2.8* *In vitro* thrombolytic efficacy

Rabbit whole blood was allowed to stand until solidification to obtain blood clots. For each experimental group, approximately 55 mg of thrombus was placed in a 12-well plate, and 3 mL of the test solution (0.5 mg/mL, corresponding to the micro/nanorobotic system or control) was added to each well. Samples were treated according to experimental conditions—exposed to a 2.0 W/cm^2^ 808 nm NIR laser and magnetic fields as required—for 1 h. After treatment, the remaining thrombus was rinsed with 1 × PBS, dried, and weighed; the thrombolysis rate was calculated by comparing the thrombus weight before and after treatment. To further evaluate thrombolytic efficacy, OD_450nm_ and OD_540nm_ were measured and used as indicators for fibrin degradation products and hemoglobin release, respectively. Additionally, hematoxylin-eosin (H&E) staining was performed to characterize changes in the internal structure of the treated thrombus.

### 2.9 In vitro hemolysis experiment

10 mL of rabbit whole blood was centrifuged at 1500 rpm for 10 min; the supernatant was discarded, and the precipitated red blood cells (RBCs) were washed with 1 × PBS and centrifuged (1500 rpm, 10 min) three times to remove residual plasma. A 5% RBC suspension was prepared by mixing 1 mL of the washed RBCs with 19 mL of 1 × PBS. Different concentrations of rGO@Fe_3_O_4_-βCD-BNN6 micro/nanorobots were prepared using 1 × PBS as the solvent. Three experimental groups were set up: a positive control group (1 mL of 5% RBC suspension mixed with 1 mL of deionized water), a negative control group (1 mL of 5% RBC suspension mixed with 1 mL of 1 × PBS), and experimental groups (1 mL of 5% RBC suspension mixed with 1 mL of rGO@Fe_3_O_4_-βCD-BNN6 micro/nanorobots of different concentrations). All groups were incubated at 37 °C for 30 min, then centrifuged at 2500 rpm for 5 min. The supernatant of each group was collected, and its OD_540nm_ was measured using a microplate reader; the hemolysis rate of rGO@Fe_3_O_4_-βCD-BNN6 micro/nanorobots was calculated based on the measured OD_540nm_ values.

### 2.10 Cytotoxicity test

The cytotoxicity of rGO@Fe_3_O_4_-βCD-BNN6 micro/nanorobots was evaluated using the CCK-8 assay, with all materials used in the experiment subjected to disinfection and sterilization prior to testing. Resuscitated human umbilical vein endothelial cells (HUVECs) were co-incubated with rGO@Fe_3_O_4_-βCD-BNN6 micro/nanorobots of different concentrations for 24 h. To investigate the photothermal-related cytotoxic effect, the experimental groups were divided into two subsets: one exposed to NIR light irradiation for 1 h and the other without NIR light exposure as a control group. After this incubation period, CCK-8 reagent was added to the cells, and the mixture was further incubated at 37 °C for 4 h to allow color development. The OD_450nm_ was measured using a microplate reader, and cell viability was determined by comparing the OD_450nm_ values of the experimental groups with those of the control group (HUVECs without material treatment) and blank group (no cells, only medium and CCK-8). To further verify the cell survival status, calcein-AM (for live cells) and propidium iodide (PI, for dead cells) were used for cell live/dead staining, and the stained cells were imaged using an inverted fluorescence microscope.

## 3 Results and discussion

### 3.1 Design and characterization of micro/nanorobots

rGO@Fe_3_O_4_ was synthesized via the coprecipitation method, where magnetic Fe_3_O_4_ nanoparticles were uniformly anchored onto the surface of rGO to endow the composite with magnetic responsiveness. To introduce binding sites for subsequent cargo loading, βCD—which contains β-cyclodextrin cavities (β sites)—was conjugated to rGO@Fe_3_O_4_, resulting in the formation of rGO@Fe_3_O_4_-βCD (a composite where rGO@Fe_3_O_4_ serves as the core and βCD provides the β sites). Subsequently, BNN6 was loaded onto rGO@Fe_3_O_4_-βCD-BNN6 micro/nanorobots through simple stirring, leveraging the host-guest interaction between BNN6 and the β sites of βCD; this process successfully yielded the final composite rGO@Fe_3_O_4_-βCD-BNN6 micro/nanorobots.

**Fig. 1:**
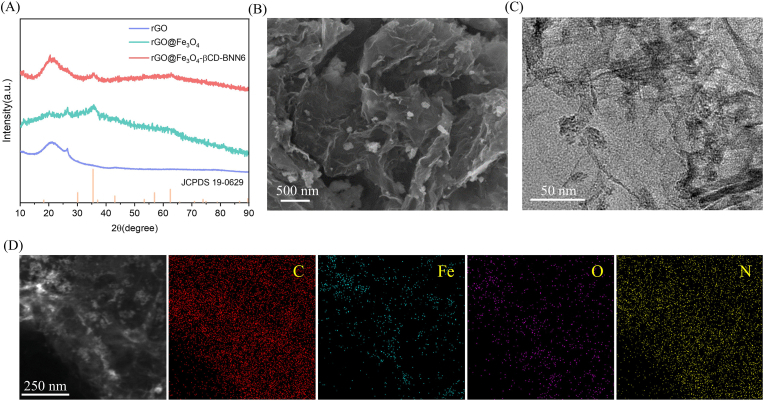
(A) XRD spectra of rGO, rGO@Fe_3_O_4,_ and rGO@Fe_3_O_4_-βCD-BNN6 micro/nanorobot. (B) SEM image of rGO@Fe_3_O_4_-βCD-BNN6 micro/nanorobots. (C) TEM image of rGO@Fe_3_O_4_-βCD-BNN6 micro/nanorobots. (D) STEM and corresponding EDS element mapping images of rGO@Fe_3_O_4_-βCD-BNN6 micro/nanorobots.

XRD was performed to characterize the crystal structure of the materials and verify the successful synthesis of the target product. As presented in Fig. 1A, although the material exhibits an amorphous nature with an ill-defined crystal structure, rGO@Fe_3_O_4_-βCD-BNN6 micro/nanorobots still show characteristic diffraction peaks corresponding to both rGO and rGO@Fe_3_O_4_. Specifically, rGO@Fe_3_O_4_ displays a distinct diffraction peak at 35.42°, assigned to the (311) crystal plane of γ-Fe_3_O_4_ [60], which confirms the successful loading of Fe_3_O_4_. Notably, rGO@Fe_3_O_4_-βCD-BNN6 micro/nanorobots also exhibit a diffraction peak at this position. This result indicates that the loading of BNN6 does not alter the crystal structure of rGO@Fe_3_O_4_, and the magnetic properties of Fe_3_O_4_ are well retained.

To further determine the surface chemical information of materials and the functional groups in their molecules, FTIR and UV-vis spectroscopy were employed to verify the successful loading of organic components. As shown in Fig. S1, rGO@Fe_3_O_4_-βCD-BNN6 micro/nanorobots share a weak characteristic peak with BNN6; however, the peak intensity is not prominent due to the amorphous structure. Additionally, the peaks in the 500-700 cm^−1^ region are attributed to the stretching vibration of Fe-O bonds [60], further confirming the successful loading of Fe_3_O_4_. More importantly, the UV spectrum of rGO@Fe_3_O_4_-βCD-BNN6 micro/nanorobots (Fig. S2) shows a distinct absorption peak at approximately 248 nm, which is the unique characteristic of BNN6.

The loading efficiency of BNN6 was determined based on the concentration of the supernatant obtained during the centrifugation process in the preparation process. For this purpose, different concentrations of BNN6 were dissolved in DMSO to establish the standard curve (Fig. S3), and the relationship between absorbance and BNN6 concentration was calibrated using the absorbance value at 371.5 nm (Fig. S4) [56]. The washing supernatant from this experiment was collected and analyzed to calculate the BNN6 loading efficiency, which was found to be 57.0% (Fig. S5). Subsequently, in vitro stability assessments were performed on the rGO@Fe_3_O_4_-βCD-BNN6 micro/nanorobots, and the results demonstrated that the micro/nanorobots maintained over 93% of its initial activity even after long-term storage, thereby confirming its excellent stability (Fig. S6).

Collectively, the above characterization results confirm the successful synthesis of rGO@Fe_3_O_4_, the effective loading of BNN6 onto rGO@Fe_3_O_4_, and thus the successful preparation of the final rGO@Fe_3_O_4_-βCD-BNN6 micro/nanorobots.

To characterize its particle size distribution of rGO@Fe_3_O_4_-βCD-BNN6 micro/nanorobots, Dynamic Light Scattering (DLS) measurements were performed. As presented in Fig. S7, the DLS results demonstrated that the particle size of the micro/nanorobots was predominantly concentrated at 356.2 nm, indicating a relatively small and uniform particle size distribution. SEM and TEM at various magnifications were used to study the surface morphology of materials, while EDS was utilized to analyze the surface element distribution. rGO is a typical two-dimensional (2D) material with a sheet-like structure [61,62]. As shown in Fig. S8, the surface of untreated rGO is smooth and presents a thin-film structure, which possesses a large surface area—this feature is favorable for the subsequent loading of other materials. After magnetization, magnetic nanoparticles were successfully loaded onto the surface of 2D rGO, as illustrated in Fig. S9. The successful loading of BNN6 is also verified by SEM images: Fig. 1B shows a significant increase in the number of particles on the 2D material surface, indicating the loading of new substances. Notably, the loading of subsequent particles does not alter the surface morphology of the pre-loaded material, which confirms the structural stability of the composite (i.e., rGO@Fe_3_O_4_-βCD-BNN6 micro/nanorobot). TEM enables clearer observation of the micro/nanorobot's microstructure. As presented in Fig. 1C and Fig. S10, TEM images at different magnifications further reveal the microscopic features of rGO@Fe_3_O_4_-βCD-BNN6 micro/nanorobots: rGO maintains a layered thin-film structure, while the loaded Fe_3_O_4_ and BNN6 exist as particles distributed on the rGO surface. To confirm that these surface particles correspond to the target chemical components, TEM elemental mapping was conducted (Fig. 1D). The results show that elements C, Fe, O, and N are uniformly distributed across the material surface. In particular, the extensive distribution of N elements further confirms the successful incorporation of BNN6 into the composite.

### 3.2 Photothermal performance of micro/nanorobots

To evaluate the photothermal properties of micro/nanorobots, the micro/nanorobots were prepared as an aqueous solution, and their temperature under NIR irradiation was measured; additionally, factors influencing photothermal performance were investigated. rGO is formed by removing partial oxygen-containing functional groups from graphene oxide (GO), which constructs a continuous conjugated π-electron system, enabling efficient absorption of light energy [63,64]. Upon photon absorption, a strong electron-phonon coupling effect occurs, and excited-state electrons rapidly convert energy into heat via non-radiative relaxation [65].

**Fig. 2:**
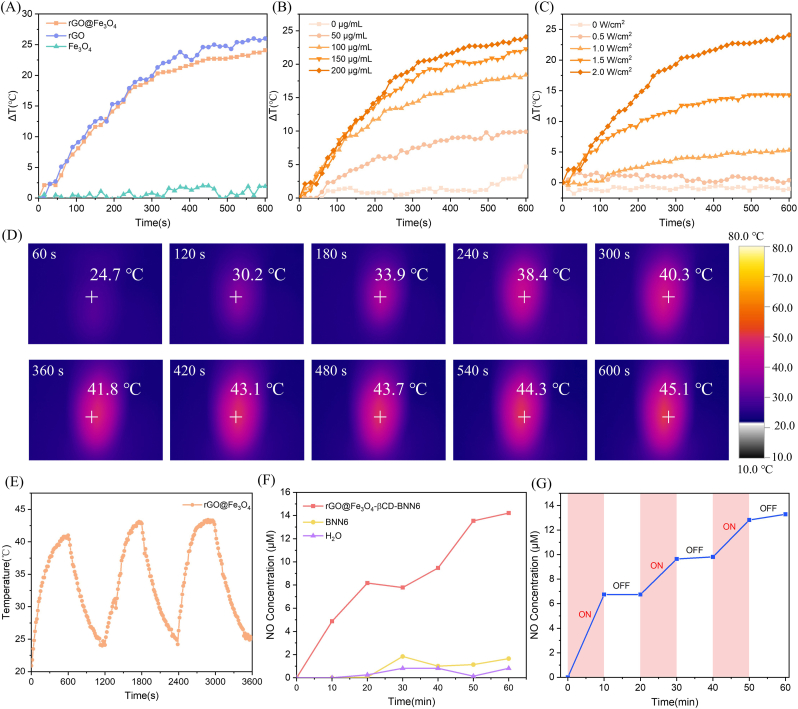
(A) Photothermal curves of 200 μg/mL rGO, Fe_3_O_4_, and rGO@Fe_3_O_4_ under 808 nm NIR laser irradiation (2.0 W/cm^2^). (B) Photothermal curves of rGO@Fe_3_O_4_ at different concentrations (0 μg/mL, 50 μg/mL, 100 μg/mL, 150 μg/mL, and 200 μg/mL) under 808 nm NIR laser irradiation (2.0 W/cm^2^). (C) Photothermal curves of 200 μg/mL rGO@Fe_3_O_4_ under irradiation of 808 nm NIR laser at different intensities (0 W/cm^2^, 0.5 W/cm^2^, 1.0 W/cm^2^, 1.5 W/cm^2^, and 2.0 W/cm^2^). (D) Infrared images of 200 μg/mL rGO@Fe_3_O_4_ under 808 nm NIR laser irradiation (2.0 W/cm^2^) at different times. (E) Photothermal stability evaluation of rGO@Fe_3_O_4_ with three cycles. (F) NO release of 0.5 mg/mL rGO@Fe_3_O_4_-βCD-BNN6 micro/nanorobots under 808 nm NIR laser irradiation (2.0 W/cm^2^). (G) Optical controllability of NO release from rGO@Fe_3_O_4_-βCD-BNN6 micro/nanorobots under switch NIR ON/OFF laser irradiation.

First, different materials (i.e., rGO, Fe_3_O_4_, and rGO@Fe_3_O_4_) were dissolved in aqueous solutions at a uniform concentration (200 μg/mL) to ensure homogeneous dispersion, thereby facilitating the photothermal property test. Each solution was exposed to 808 nm NIR irradiation (2.0 W/cm^2^) for 10 min, with temperature recorded every 15 s. As shown in Fig. 2A, the temperature variation (Δ*T*) of pure rGO within 10 min reached 26.0 °C, confirming its excellent photothermal conversion performance. In contrast, Fe_3_O_4_ (a magnetic material) lacks such photothermal activity—its temperature curve remained nearly stable, leading to a slight reduction in the photothermal performance of rGO@Fe_3_O_4_ (maximum (Δ*T*: 2.1 °C).

Both solution concentration and laser irradiation intensity affected the photothermal temperature: as the solution concentration increased (from 50 μg/mL to 200 μg/mL) and the laser intensity strengthened (from 0.5 W/cm^2^ to 2 W/cm^2^), the photothermal temperature of rGO@Fe_3_O_4_ increased correspondingly (Fig. 2B and. C), with actual infrared temperature measurement image of the solution is shown in Fig. 2D. Notably, rGO@Fe_3_O_4_ still maintained a sufficiently high photothermal temperature for potential application in photothermal therapy. The photothermal effect stability of rGO@Fe_3_O_4_ was a key indicator. After three cycles of NIR laser irradiation on-off testing, rGO@Fe_3_O_4_ retained stable photothermal performance: under NIR irradiation, the temperature increased steadily, while a certain period was required for temperature recovery to return to the initial state after NIR was turned off—indicating favorable recyclability and stability (Fig. 2E). The photothermal conversion efficiency of rGO@Fe_3_O_4_ was calculated to be 44.2%, further validating its excellent photothermal performance (Figure S11 and 12).

The aforementioned photothermal tests confirmed that rGO@Fe_3_O_4_ has excellent thermal conversion capability. Based on this, BNN6 was loaded onto rGO@Fe_3_O_4_, with the aim of utilizing the heat generated under NIR irradiation to promote BNN6 decomposition and NO production. The concentration of NO generated by rGO@Fe_3_O_4_-βCD-BNN6 micro/nanorobots under 808 nm NIR irradiation was quantitatively analyzed via the Griess method. As shown in Fig. 2F and 0.5 mg/mL rGO@Fe_3_O_4_-βCD-BNN6 micro/nanorobots produced NO under 808 nm NIR laser irradiation (2.0 W/cm^2^), with NO concentration increasing gradually over time. To verify the controllability of NO production, cyclic on-off NIR irradiation was applied: NO production increased rapidly when NIR was on, while negligible NO generation was observed when NIR was off (Fig. 2G). To preclude the premature leakage of rGO@Fe_3_O_4_-βCD-BNN6 micro/nanorobots during therapeutic applications, an incubation assay was conducted at 37 °C. Following an incubation period of 1 h, no significant alterations in the stability of the material were observed, with the micro/nanorobots retaining 90% of their initial activity, thus corroborating their favorable stability under physiological-mimetic conditions (Fig. S14). This result demonstrates the light-controllable NO behavior of rGO@Fe_3_O_4_-βCD-BNN6 micro/nanorobots, enabling precise regulation of NO release and cessation simply by switching NIR irradiation on or off in subsequent application.

### 3.3 Motion behaviors of rGO@Fe_3_O_4_ micro/nanorobots

**Fig. 3:**
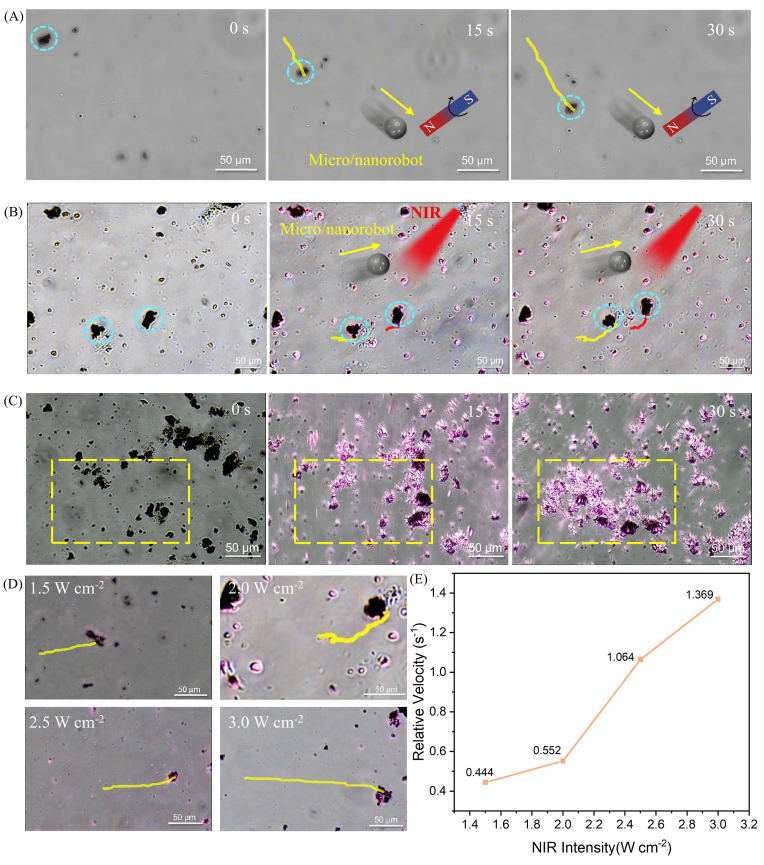
(A) Time-lapse images of rGO@Fe_3_O_4_ micro/nanorobots under a rotating magnetic field. (B) Time-lapse images of rGO@Fe_3_O_4_ micro/nanorobots upon irradiation with an 808 nm NIR laser at a power density of 2.0 W/cm^2^. (C) Time-lapse images illustrating the light-induced collective behavior of rGO@Fe_3_O_4_ micro/nanorobots under irradiation with an 808 nm NIR laser at a power density of 2.0 W/cm^2^. (D) Time-lapse images of rGO@Fe_3_O_4_ micro/nanorobots under irradiation with an 808 nm NIR laser at different power densities (1.5 W/cm^2^, 2.0 W/cm^2^, 2.5 W/cm^2^, and 3.0 W/cm^2^). (E) Plot showing the relationship between the relative velocity of rGO@Fe_3_O_4_ micro/nanorobots and the intensity of 808 nm NIR laser irradiation.

The motion characteristics of micro/nanorobots are among their key features, and their locomotive behaviors can be observed via an inverted fluorescence microscope. Owing to the incorporated magnetic Fe_3_O_4_ particles, the rGO@Fe_3_O_4_ micro/nanorobots exhibit rapid rolling motion under a rotating magnetic field (RMF) (Fig. 3A, Video S1). This observation not only confirms the successful loading of Fe_3_O_4_ but also provides experimental support for the magnetic-driven motion of the micro/nanorobots.

Notably, the rGO@Fe_3_O_4_ micro/nanorobots can achieve motion under NIR irradiation, which is attributed to the excellent photothermal performance of rGO. Fig. 3B presents time-lapse images of the trajectory of rGO@Fe_3_O_4_ micromotors under NIR irradiation; after 30 s, the particles show significant displacement relative to their initial positions (**Vedio S2**). To further explore whether rGO@Fe_3_O_4_ micro/nanorobots possess light-induced collective behaviors, a localized NIR light spot was applied to the sample. As shown in Fig. 3C, the rGO@Fe_3_O_4_ micro/nanorobots aggregate in the specific region marked by the yellow square (**Vedio S3**). This phenomenon clearly demonstrates the light-aggregation effect of the rGO@Fe_3_O_4_ material, which is beneficial for enhancing its photothermal properties, enabling targeted therapy, and improving both the material utilization rate and treatment efficiency.

To investigate the influence of light intensity on the movement speed of particles, the rGO@Fe_3_O_4_ micro/nanorobots were placed in the NIR region with different intensities, and their motion rates within a 30-s interval were recorded. The results show that as the NIE intensity increases, the moving distance of the micro/nanorobots within the same period gradually increases (Fig. 3D–Videos S4-6). During the experiment, it was also observed that particles with larger volumes exhibited faster motion speeds. To eliminate the influence of particle size on the evaluation of motion performance, “relative velocity” (defined as the ratio of speed to body length [66]) was introduced. As shown in Fig. 3E, the relative velocity of rGO@Fe_3_O_4_ micro/nanorobots increases approximately linearly with the increase in laser irradiation intensity.

Given the intended application of the composite in thrombolytic therapy, its locomotor behavior in blood matrices warrants investigation. Under the stimulation of a rotating magnetic field, rGO@Fe_3_O_4_ micro/nanorobots maintained continuous rotational propulsion in blood, indicating that the complex components present in blood do not compromise the locomotor activity of the composite (Video S7). Furthermore, under NIR light irradiation, rGO@Fe_3_O_4_ micro/nanorobots retained their locomotor capability in blood environments (Video S8). Collectively, these findings demonstrate that rGO@Fe_3_O_4_ micro/nanorobots can achieve effective locomotion not only in aqueous media but also in complex blood matrices, thereby validating the superior locomotor performance of the fabricated micro/nanorobots.

In summary, the motion of rGO@Fe_3_O_4_ micro/nanorobots can be jointly regulated by magnetic and light fields: the magnetic field enables precise control over the motion direction, while the light field provides the main driving force and induces particle aggregation in specific areas, thereby realizing efficient photothermal conversion.

### 3.4 The effects of extracorporeal thrombolysis and its synergistic mechanism

Given the excellent photothermal conversion performance and motion behavior of rGO@Fe_3_O_4_-βCD-BNN6 micro/nanorobots, they can be applied for photothermal thrombolysis. To evaluate their thrombolytic efficacy, blood clots were subjected to eight different treatment groups: (1) PBS, (2) rGO@Fe_3_O_4_, (3) rGO@Fe_3_O_4_-βCD-BNN6, (4) UK, (5) PBS + NIR, (6) rGO@Fe_3_O_4_+NIR, (7) rGO@Fe_3_O_4_-βCD-BNN6+NIR, (8) rGO@Fe_3_O_4_-βCD-BNN6+NIR + RMF. All experiments were conducted for a duration of 1 h.

**Fig. 4:**
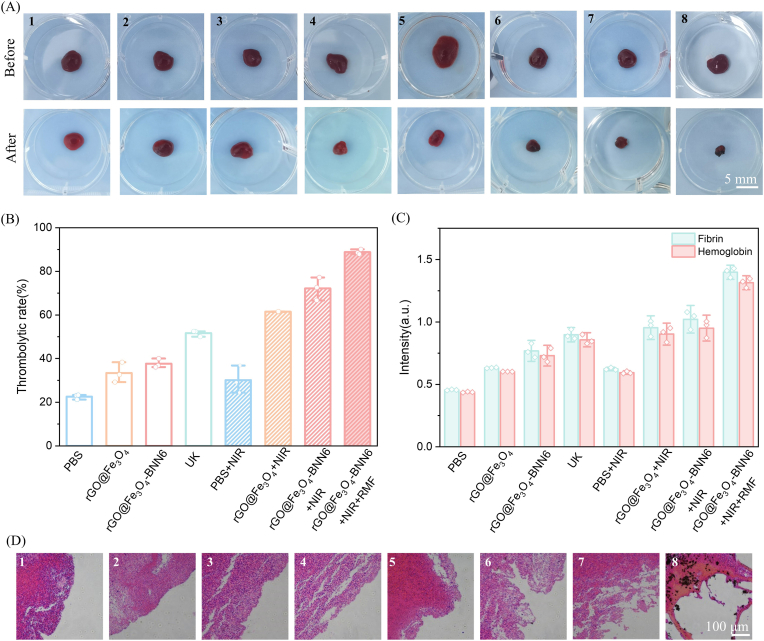
(A) Comparison of thrombus clot before and after 1 h of treatment. (B) Thrombolysis rates of thrombi under different treatment conditions. (C) Levels of fibrin and hemoglobin in the supernatant of thrombi after different treatments. (D) H&E staining images of thrombi subjected to different treatments. Notes: (1) PBS, (2) rGO@Fe_3_O_4_, (3) rGO@Fe_3_O_4_-βCD-BNN6, (4) UK, (5) PBS + NIR irradiation, (6) rGO@Fe_3_O_4_+NIR irradiation, (7) rGO@Fe_3_O_4_-βCD-BNN6+NIR irradiation, and (8) rGO@Fe_3_O_4_-βCD-BNN6+NIR irradiation + RMF.

Among these groups, the rGO@Fe_3_O_4_-βCD-BNN6+NIR + RMF treatment resulted in a significant reduction in thrombus volume (Fig. 4A). Thrombus ablation rates were calculated based on the thrombus mass before and after the treatment, and the results for each group are presented in Fig. 4B. Notably, 808 nm NIR irradiation markedly enhanced the thrombolysis rate: treatment with rGO@Fe_3_O_4_ micro/nanorobots alone achieved a 33.4% ablation rate, while the addition of NIR irradiation increased this rate to 61.5% (nearly double). Furthermore, the incorporation of BNN6 led to NO generation via the photothermal effect, which further promoted thrombus dissolution and elevated the thrombolysis rate to 72.2%. Under NIR laser irradiation, the photothermal properties of rGO@Fe_3_O_4_-βCD-BNN6 micro/nanorobots caused the local temperature to rise to 42.3 °C (Fig. S15). When an RMF was applied, the targeted motion of the micro/nanorobots was enhanced, enabling more thorough thrombus treatment and increasing the thrombolysis rate to 88.8%—accompanied by a final thrombus weight of 6.2 mg. This efficacy was substantially superior to that of the clinical drug UK, which only achieved a 51.6% thrombolysis rate, highlighting rGO@Fe_3_O_4_-βCD-BNN6 micro/nanorobots as a promising alternative for physical thrombolytic therapy.

These results confirm the feasibility of rGO@Fe_3_O_4_-βCD-BNN6 as the photothermal thrombolytic material of micro/nanorobots: NO generated by the photothermal decomposition of loaded BNN6 contributes additional thrombolytic activity, while the micro/nanorobots’ motion performance further boosts the thrombolysis rate. The OD of the supernatant was also used to characterize thrombolytic performance, as thrombus dissolution releases hemoglobin and fibrin (measured by OD_415nm_ and OD_540nm_, respectively). As shown in Fig. 4C, the OD results were consistent with the thrombolysis rate data.

Collectively, the aforementioned findings corroborate that thrombus ablation is primarily mediated by the photothermal effect of the composite and the release of NO. To validate the synergistic significance of this dual therapeutic mechanism, experimental Groups 9 (Fe_3_O_4_-βCD-BNN6) and 10 (Fe_3_O_4_-βCD-BNN6+NIR) were established. In the absence of rGO, the composite exhibited negligible photothermal responsiveness, with thrombus treatment efficiencies of merely 38.2% and 50.7%, respectively (Fig. S16; Fig. S17). To further elucidate the pivotal role of NO in thrombolysis, Group 11 (rGO@Fe_3_O_4_-βCD-BNN6+NO scavenger + NIR) was constructed to deplete endogenous NO and evaluate its independent contribution to thrombus degradation. Upon NO scavenging, the thrombolysis rate was reduced to 56.9%, which provides indirect evidence for the critical involvement of NO in the therapeutic efficacy against thrombi (Fig. S16; Fig. S17). Additionally, the absorbance values (OD_450 nm_ and OD_540 nm_) of the supernatant were consistent with the aforementioned thrombolysis patterns, further substantiating that the synergistic effects of photothermal therapy (PTT) and NO release facilitate efficient thrombus resolution (Fig. S18).

H&E staining was employed to assess changes in thrombus microstructure. Thrombi treated with PBS (control group) showed almost no damage, consistent with their low thrombolysis rate. In contrast, thrombi treated with rGO@Fe_3_O_4_-βCD-BNN6 micro/nanorobots under the combined action of RMF and NIR exhibited significant surface damage, further validating the thrombolytic efficacy of micro/nanorobots. The cross-sectional results for the Fe_3_O_4_-βCD-BNN6 and Fe_3_O_4_-βCD-BNN6+NIR groups shown in Fig. S19 indicate that they exhibit poor thrombus lysis efficiency, consistent with the aforementioned results, and further demonstrate the synergistic effect of photothermal action and NO-mediated promotion. In contrast, the results for the rGO@Fe_3_O_4_-βCD-BNN6+NO scavenger + NIR group were significantly worse than those for the rGO@Fe_3_O_4_-βCD-BNN6+NIR group, further highlighting the crucial role of NO in this system. A close examination of Groups 7 (rGO@Fe_3_O_4_-βCD-BNN6+NIR) and 8 (rGO@Fe_3_O_4_-βCD-BNN6+NIR + RMF) in Fig. 4D reveals that, even after the addition of RMF, numerous black particles remain within the treated thrombus. This serves as direct evidence that the rGO@Fe_3_O_4_-βCD-BNN6 micro/nanorobots have penetrated the thrombus to deliver deep-tissue therapy. Therefore, during this therapeutic process, the presence of a magnetic field effectively induces movement and agitation within the material, enabling deep-tissue and mechanical agitation therapy to break down the thrombus structure.

To further characterize the degradation behavior of the composite post-treatment, UV-Vis spectroscopy was employed to evaluate the degradation of rGO@Fe_3_O_4_-βCD-BNN6 micro/nanorobots before and after the therapeutic intervention. As illustrated in Fig. S20, approximately 20% of the rGO@Fe_3_O_4_-βCD-BNN6 micro/nanorobots underwent degradation following 1 h of NIR irradiation, indicating structural disruption of the composite during the therapeutic process. Collectively, the integrated properties of rGO@Fe_3_O_4_-βCD-BNN6 micro/nanorobots —including photothermal conversion, magnetically controlled locomotion, and controlled NO generation—enable robust thrombus ablation with superior thrombolytic efficacy. While the in vitro thrombolysis assays comprehensively demonstrated the exceptional performance of the composite, this work remains in the proof-of-concept stage. Future investigations will focus on the utilization of microfluidic devices to explore the translational potential of the fabricated micro/nanorobots in clinical thrombolytic therapy.

### 3.5 Biocompatibility analysis

**Fig. 5:**
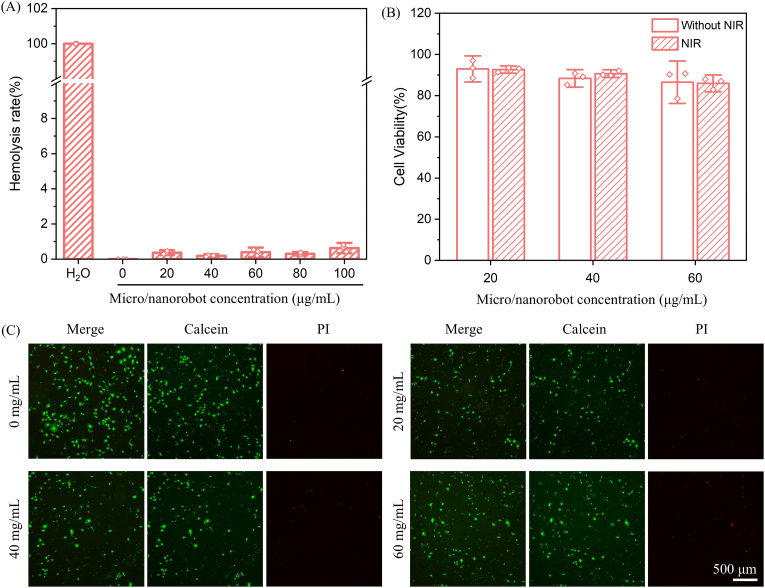
(A) Hemolysis rate of red blood cells treated with water (positive control) and rGO@Fe_3_O_4_-βCD-BNN6 micro/nanorobots at different concentrations. (B) Relative viability of HUVECs treated with rGO@Fe_3_O_4_-βCD-BNN6 micro/nanorobots at various concentrations for 24 h, measured by the CCK-8 assay. (C) Live/dead staining analysis of HUVECs co-cultured with rGO@Fe_3_O_4_-βCD-BNN6 micro/nanorobots at different concentrations for 24 h; green fluorescence indicates live cells, while red fluorescence indicates dead cells. (For interpretation of the references to color in this figure legend, the reader is referred to the Web version of this article.)

Biocompatibility assessment is essential for the biomedical application of any material, and rGO@Fe_3_O_4_-βCD-BNN6 micro/nanorobots were evaluated systematically in this regard. Blood compatibility was assessed via hemolysis assays, where different concentrations of rGO@Fe_3_O_4_-βCD-BNN6 micro/nanorobots were tested. As shown in Fig. 5A, the hemolysis rate of all tested concentrations remained below 5%, confirming excellent blood compatibility of micro/nanorobots. Anticoagulant activity assays were performed to assess the potential adverse effects of the composite on the blood microenvironment. The results indicated that, in comparison with the control group, the activated partial thromboplastin time (APTT) for rGO@Fe_3_O_4_-βCD-BNN6 micro/nanorobot group did not exhibit a statistically significant increase. This finding demonstrates that the composite exerts minimal perturbation on the coagulation system, thereby confirming its favorable anticoagulant performance (Fig. S21). HUVECs are commonly used in cell viability assays to evaluate material safety. After incubating HUVECs with rGO@Fe_3_O_4_-βCD-BNN6 micro/nanorobots at various concentrations for 24 h, the cells maintained high viability (all above 80%), as illustrated in Fig. 5B. Furthermore, under NIR irradiation, HUVECs maintained similar levels of cellular activity, demonstrating the safety of the material. To further verify the non-toxicity of rGO@Fe_3_O_4_-βCD-BNN6 micro/nanorobots, live/dead staining of HUVECs was performed using calcein-AM (which emits green fluorescence for live cells) and PI (which emits red fluorescence for dead cells), with observations made via an inverted fluorescence microscope. As presented in Fig. 5C, most cells in the images exhibited strong green fluorescence signals, while only a few showed weak red fluorescence. This indicates that the HUVECs incubated with different concentrations of rGO@Fe_3_O_4_-βCD-BNN6 micro/nanorobots remained viable, confirming the non-toxicity and favorable biocompatibility for potential biomedical applications.

## 4 Conclusions

In summary, we have developed a novel dual-driven (magnetic/photothermal) rGO@Fe_3_O_4_-βCD-BNN6 micro/nanorobots platform for targeted thrombolysis. This system capitalizes on the excellent photothermal conversion performance of rGO@Fe_3_O_4_: upon 808 nm NIR laser irradiation, it induces rapid local hyperthermia to mediate effective photothermal thrombolysis, while concurrently triggering thermal decomposition of the loaded NO donor BNN6. The in-situ released NO further augments thrombolytic efficacy by mechanically disrupting fibrin networks. Notably, under the RMF stimulation, the micro/nanorobots exhibit precise directional locomotion and undergo collective aggregation in NIR-irradiated regions—a feature that not only enhances thrombolytic efficiency but also enables the treatment of deep-seated thrombi. Systematic in vitro biocompatibility evaluations confirm the excellent safety profile of the micro/nanorobots. Collectively, the rGO@Fe_3_O_4_-βCD-BNN6 micro/nanorobots enable synergistic, targeted, and safe photothermal-NO thrombolysis, providing a promising avenue for advancing micro/nanorobotic-based thrombolytic therapies.

## CRediT authorship contribution statement

**Wenjia Kang:** Conceptualization, Data curation, Formal analysis, Investigation, Methodology, Writing – original draft. **Jinhua Li:** Conceptualization, Funding acquisition, Resources, Supervision, Writing – review & editing. **Song Li:** Investigation. **Yingting Yang:** Investigation. **Shanqing Gao:** Investigation. **Shuangying Wei:** Investigation. **Zdenek Sofer:** Resources. **Jiatao Zhang:** Resources, Supervision. **Huaijuan Zhou:** Conceptualization, Funding acquisition, Project administration, Resources, Supervision, Visualization, Writing – original draft, Writing – review & editing.

## Declaration of competing interest

The authors declare that they have no known competing financial interests or personal relationships that could have appeared to influence the work reported in this paper.

## Data Availability

Data will be made available on request.
