# Supplementary material for: Magnetic/photothermal dual-driven micro/nanorobots for synergistic NO-mediated photothermal thrombolysis
Source: Mater Today Bio. 2026 Apr 30;38:103179. doi: 10.1016/j.mtbio.2026.103179 (PMC13153597; doi:10.1016/j.mtbio.2026.103179)
Supplement: Multimedia component 1 [file mmc1.docx]

**Supplementary Information**

**Magnetic/photothermal dual-driven micro/nanorobots for synergistic NO-mediated photothermal thrombolysis**

Wenjia Kang^1^, Jinhua Li^2,^ *, Song Li^1^, Yingting Yang^1^, Shanqing Gao^2^, Shuangying Wei^3^, Zdenek Sofer^4^, Jiatao Zhang^2,^ *, Huaijuan Zhou^1,^ *

^1^ School of Materials Science and Engineering, School of Interdisciplinary Science, Beijing Institute of Technology, Beijing 100081, China

^2^ Key Laboratory of Medical Molecule Science and Pharmaceutical Engineering, Ministry of Industry and Information Technology, Beijing Key Laboratory of Intelligent Molecular Materials and High-throughput Manufacturing, School of Chemistry and Chemical Engineering, Beijing Institute of Technology, Beijing, 100081, China.

^3^ Department of Industrial Chemistry, University of Bologna, Campus Navile, Via Piero Gobetti 85, Bologna 40139, Italy

^4^ Department of Inorganic Chemistry, University of Chemistry and Technology Prague, Technicka 5, 166 28, Prague 6, Czech Republic

*Corresponding authors:

E-mail address: [lijinhua@bit.edu.cn](mailto:lijinhua@bit.edu.cn) (J. Li), [zhangjt@bit.edu.cn](mailto:zhangjt@bit.edu.cn) (J. Zhang), and [huaijuan.zhou@bit.edu.cn](mailto:huaijuan.zhou@bit.edu.cn) (H. Zhou)

**1. Experimental section**

**1.1 Synthesis of rGO**

Reduced graphene oxide (rGO) was synthesized via a modified Hummers method [1,2]. Natural graphite (99.99%, Alpha Aesar) was used as the raw material to prepare a colloidal graphite suspension.. Graphite particles with a size range of 2–15 μm were oxidized using concentrated sulfuric acid, phosphoric acid, potassium permanganate, and hydrogen peroxide. The resulting mixture was formulated into a 2.5 mg/mL suspension, which was then centrifuged and vacuum-filtered through a 0.45 μm polycarbonate (PC) membrane (Nukoper, Φ90 mm) to produce graphite oxide foil. The GO foil was peeled off the PC membrane after drying at 50 °C for several hours, followed by heat treatment at 600 °C in a mixed H₂/N₂ atmosphere for 2 hours to yield rGO foil.

**1.2 Stability verification of rGO@Fe_3_O_4_-βCD-BNN6 micro/nanorobots**

Freshly prepared rGO@Fe_3_O_4_-βCD-BNN6 micro/nanorobots were stored at -20°C in the dark. After 14 days of storage, the stability of the micro/nanorobots was evaluated by comparing it with freshly prepared rGO@Fe_3_O_4_-βCD-BNN6 mirorobots using UV-Vis spectroscopy. Additionally, the temperature stability of of rGO@Fe_3_O_4_-βCD-BNN6 micro/nanorobots was assessed by incubating its solution in a 37°C water bath for 1 hour, followed by UV-Vis spectroscopic comparison with the freshly prepared ones.

**1.3 Degradation of rGO@Fe_3_O_4_-βCD-BNN6**

To investigate the degradation behavior of rGO@Fe_3_O_4_-βCD-BNN6 micro/nanorobots before and after the thrombolysis experiment, the micro/nanorobots were collected post-experiment, then thoroughly washed, centrifuged, and dried. A solution was prepared using the treated micro/nanorobots, and its UV-Vis spectrum was compared with that of the original micro/nanorobots to evaluate the degradation degree of rGO@Fe_3_O_4_-βCD-BNN6 micro/nanorobots.

**1.4 Anticoagulant properties of rGO@Fe_3_O_4_-βCD-BNN6 micro/nanorobots**

Rabbit whole blood was centrifuged at 3000 r/min for 15 minutes, and the plasma was collected from the supernatant. Two groups were established: a normal control group (pure plasma) and an experimental group (plasma supplemented with rGO@Fe_3_O_4_-βCD-BNN6 micro/nanorobots). After equilibrating the activated partial thromboplastin time (APTT) tannic acid solution to room temperature, 0.1 mL of the test plasma and 0.1 mL of the tannic acid solution were pipetted, mixed thoroughly, and incubated in a 37 ^o^C water bath for 5 minutes with gentle mixing at intervals. Subsequently, 0.1 mL of pre-incubated CaCl_2_ solution (25 mM, 37 ^o^C) was added to the mixture. The timer was started immediately, and the mixture was placed in the water bath with continuous shaking. The time required for the appearance of fibrin strands was recorded. Each experiment was repeated twice, and the average value was calculated.

**Figure S1.** FTIR spectra of rGO, rGO@Fe_3_O_4_, rGO@Fe_3_O_4_-βCD-BNN6 and BNN6.

**Figure S2.** UV-Vis spectra of rGO@Fe_3_O_4_, rGO@Fe_3_O_4_-βCD-BNN6 and BNN6.

**Figure S3.** UV spectra of BNN6 of different concentrations.

**Figure S4.** Standard curve of absorbance at the wavelength of 371.5 nm for BNN6.

**Figure S5.** UV absorption spectrum of the supernatant of rGO@Fe_3_O_4_-βCD-BNN6 micro/nanorobots.

**Figure S6.** The stability of rGO@Fe_3_O_4_-βCD-BNN6 micro/nanorobots stored for 14 days.

**Figure S7.** DLS test of rGO@Fe_3_O_4_-βCD-BNN6 micro/nanorobots.

**
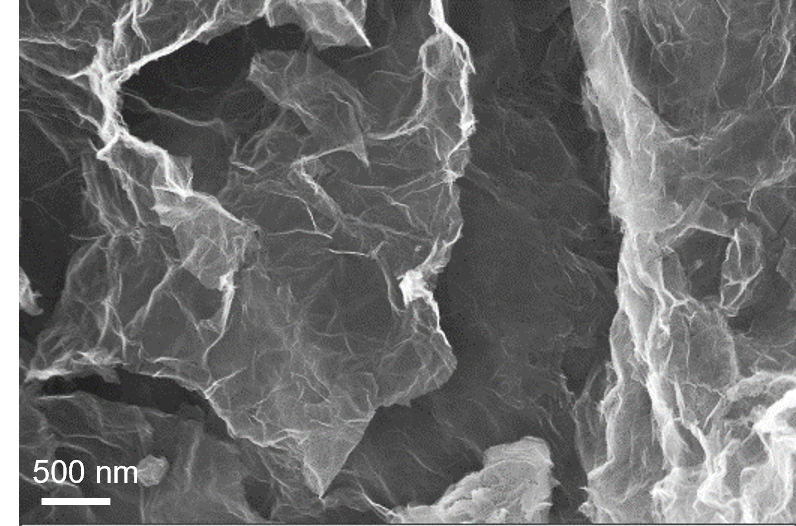
**

**Figure S8.** SEM image of rGO.

**
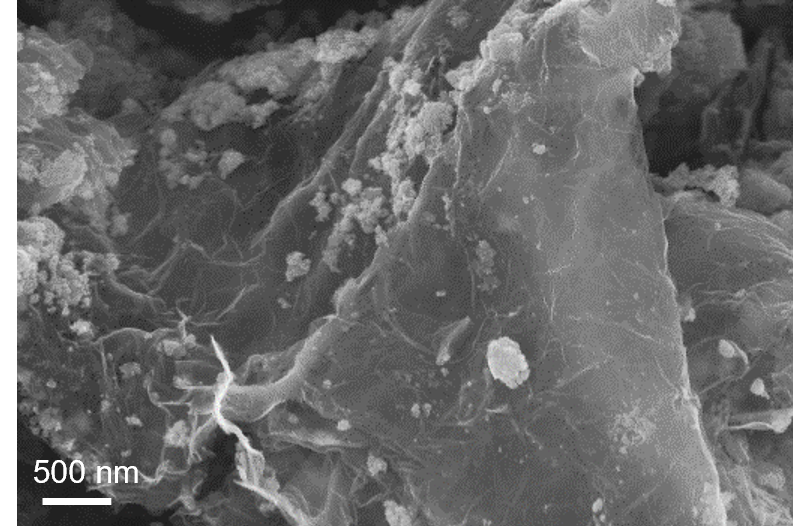
**

**Figure S9.** SEM image of rGO@Fe_3_O_4_.

**
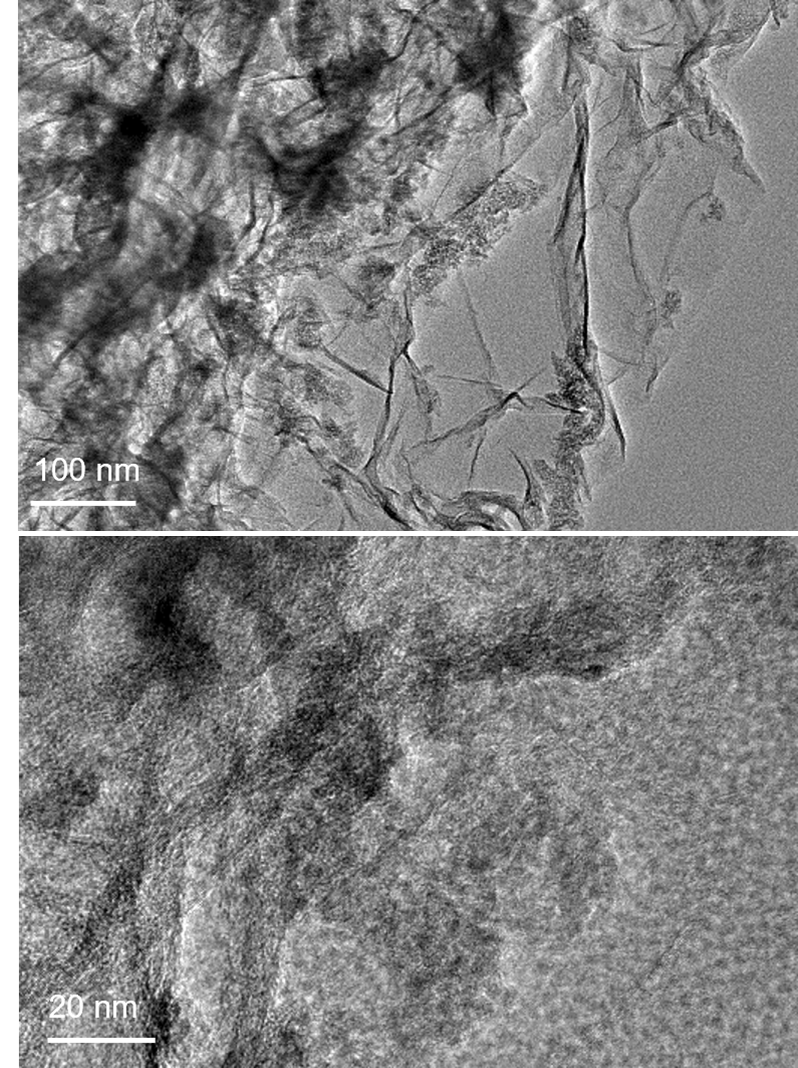
**

**Figure S10.** TEM images of rGO@Fe_3_O_4_-βCD-BNN6 micro/nanorobots at different magnification times.

**Figure S11.** Heating curves of rGO@Fe_3_O_4_ and H_2_O under 808 nm NIR laser and cooling curves after laser off.

**Figure S12.** Fitting graph of the cooling time of rGO@Fe_3_O_4_ and -*ln*(θ).

**Figure S13.** Calibration curve of the absorbance of NaNO_2_ at 540 nm.

**Figure S14.** Stability of rGO@Fe_3_O_4_-βCD-BNN6 micro/nanorobots after 1 h incubation at 37 °C.


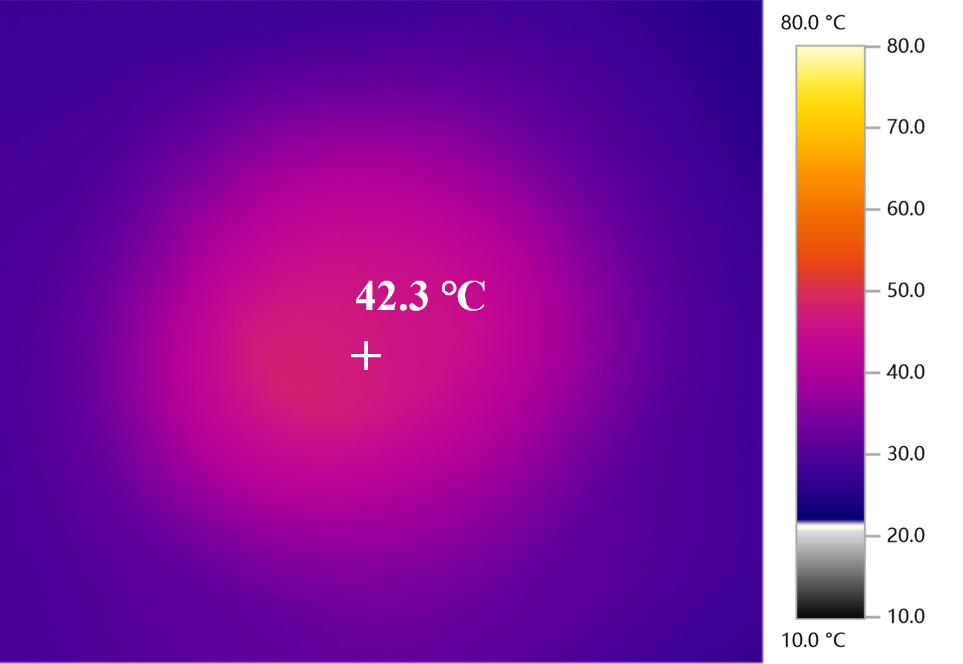


**Figure S15.** Infrared thermometry images of rGO@Fe_3_O_4_-βCD-BNN6 micro/nanorobots during thrombosis treatment.


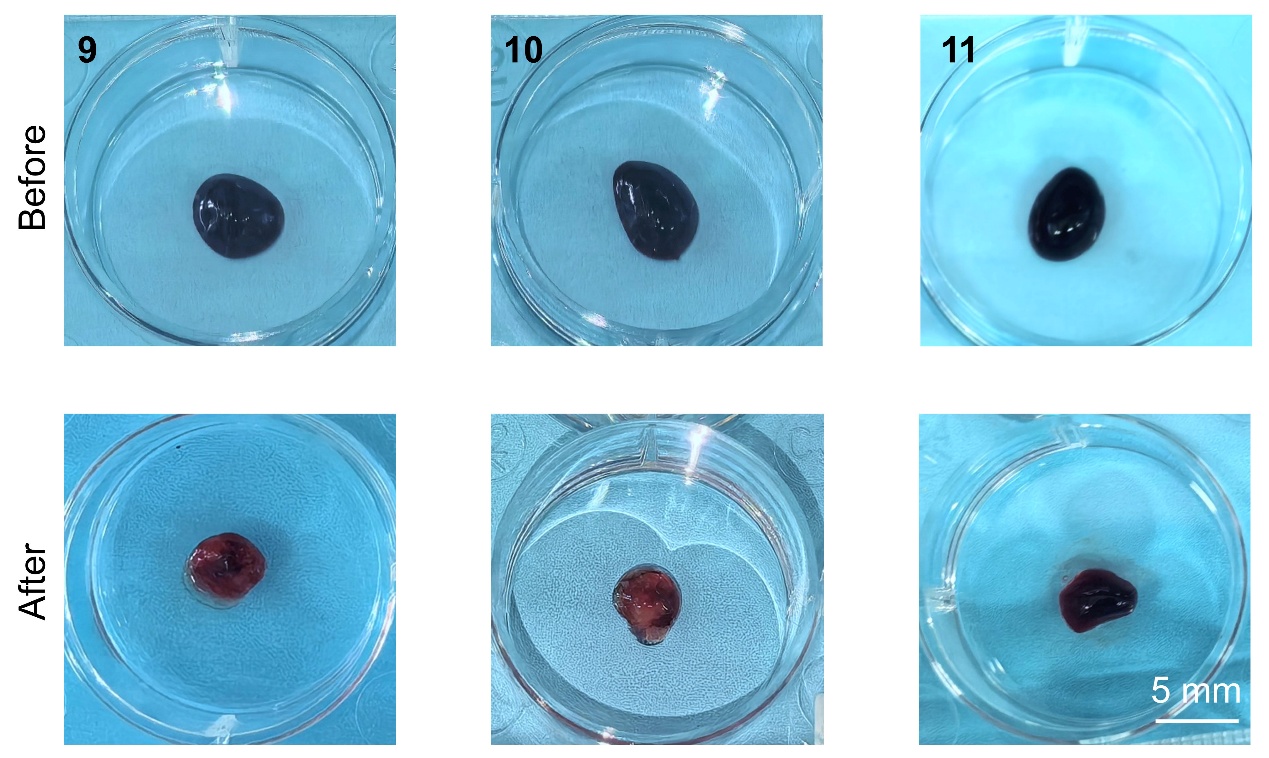


**Figure S16.** Comparison of thrombus clot before and after 1 hour of treatment (Group 9: Fe_3_O_4_-βCD-BNN6; Group 10: Fe_3_O_4_-βCD-BNN6+NIR; Group 11: rGO@Fe_3_O_4_-βCD-BNN6 micro/nanorobots+NO scavenger+NIR).

**Figure S17.** Thrombolysis rates of thrombi under different treatment conditions.

**Figure S18.** Levels of fibrin and hemoglobin in the supernatant of thrombi after different treatments.


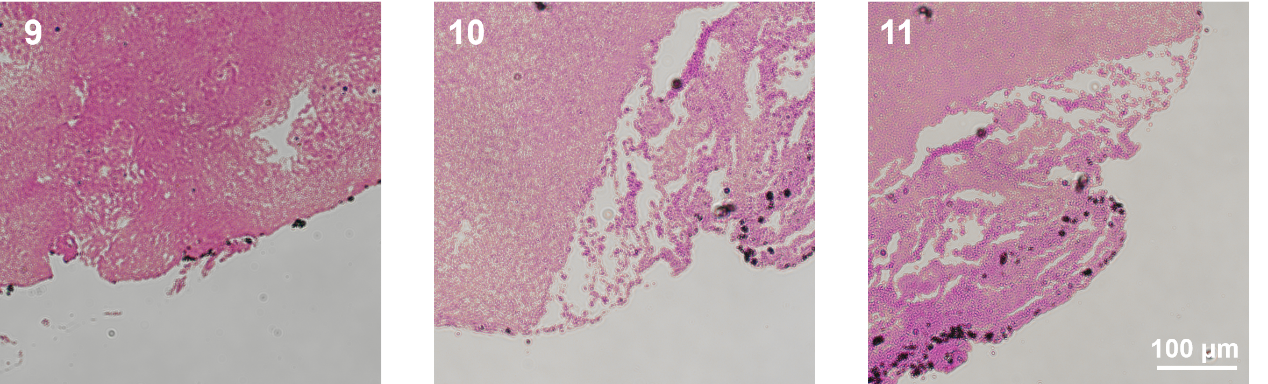


**Figure S19.** H&E staining images of thrombi subjected to different treatments. Group 9: Fe_3_O_4_-βCD-BNN6; Group 10: Fe_3_O_4_-βCD-BNN6+NIR irradiation; Group 11: rGO@Fe_3_O_4_-βCD-BNN6 micro/nanorobots+NO scavenger+NIR irradiation.

**Figure S20.** Degradation of rGO@Fe_3_O_4_-βCD-BNN6 micro/nanorobots after thrombus removal.

**Figure S21.** Anticoagulant properties of rGO@Fe_3_O_4_-βCD-BNN6 micro/nanorobots.

**References**

[1] W.S. Hummers, Jr., and R.E. Offeman, Preparation of Graphitic Oxide, Journal of the American Chemical Society 80 (1958) 1339-1339.

[2] F. Zoller, D. Böhm, J. Luxa, M. Döblinger, Z. Sofer, D. Semenenko, T. Bein, D. Fattakhova-Rohlfing, Freestanding LiFe_0.2_Mn_0.8_PO_4_/rGO nanocomposites as high energy density fast charging cathodes for lithium-ion batteries, Materials Today Energy 16 (2020) 100416.
